# Epigenetic clocks suggest accelerated aging in patients with isolated REM Sleep Behavior Disorder

**DOI:** 10.1038/s41531-023-00492-2

**Published:** 2023-03-30

**Authors:** Luca Baldelli, Chiara Pirazzini, Luisa Sambati, Francesco Ravaioli, Davide Gentilini, Giovanna Calandra-Buonaura, Pietro Guaraldi, Claudio Franceschi, Pietro Cortelli, Paolo Garagnani, Maria Giulia Bacalini, Federica Provini

**Affiliations:** 1 Department of Biomedical and NeuroMotor Sciences (DiBiNeM), University of Bologna, Bologna, Italy; 2 IRCCS Istituto delle Scienze Neurologiche di Bologna, Bologna, Italy; 3 Department of Medical and Surgical Sciences (DIMEC), University of Bologna, Bologna, Italy; 4 Department of Brain and Behavioral Sciences, University of Pavia, Pavia, Italy; 5 Bioinformatics and Statistical Genomics Unit, Istituto Auxologico Italiano IRCCS, Cusano Milanino, Milan, Italy; 6 Institute of Information Technologies, Mathematics and Mechanics, Lobachevsky State University, Nizhny Novgorod, Russia

**Keywords:** Neurodegeneration, Predictive markers, Parkinson's disease

## Abstract

Isolated REM Sleep Behavior Disorder (iRBD) is the strongest prodromal marker for α-synucleinopathies. Overt α-synucleinopathies and aging share several mechanisms, but this relationship has been poorly investigated in prodromal phases. Using DNA methylation-based epigenetic clocks, we measured biological aging in videopolysomnography confirmed iRBD patients, videopolysomnography-negative and population-based controls. We found that iRBDs tended to be epigenetically older than controls, suggesting that accelerated aging characterizes prodromal neurodegeneration.

## Introduction

Isolated REM Sleep Behavior Disorder (iRBD) is a well-recognized prodromal state of an underlying α-synucleinopathy, occurring several years before the majority of patients convert to an overt neurodegenerative disorder. The presence of iRBD indicates that neurodegeneration of specific brainstem nuclei has already started. Indeed, these patients can manifest subtle motor signs and a variety of non-motor symptoms (NMS), reflecting the increase of the neurodegenerative burden^1^.

Advanced age is a major risk factor for neurodegenerative diseases and the existence of a continuum between aging and α-synucleinopathies has been proposed^2^. However, little is known about the impact of aging on iRBD and whether these patients are biologically older than their chronological age.

Among the most reliable biomarkers of age are the so-called epigenetic clocks: mathematical models that, starting from DNA methylation profiles of different sets of CpG dinucleotide sites, return an estimate of the age of an individual^3,4^. The discrepancy between predicted epigenetic age and chronological age (i.e. the epigenetic age acceleration—EAA) has proven to be informative of biological age in several pathological conditions, including PD^5^.

In this paper, we used different types of epigenetic clocks to compare epigenetic age between videopolysomnography (vPSG)-confirmed iRBD patients (iRBDs), vPSG-negative controls (CTR_neg) and controls from the general population (CTR_pop).

## Results

### Demographics and clinical follow-up

We compared 28 iRBDs (23 males, age 67.92 ± 7.20 years), 57 CTR_neg (32 males, age 66.56 ± 9.56 years) and 31 CTR_pop (15 males, age 68.25 ± 7.23 years); male prevalence was higher among iRBDs (*p* = 0.020), age was comparable (*p* = 0.614). iRBDs presented a mean disease duration of 8.95 ± 6.35 years (range 1–28 years) and were followed up for a mean of 3.45 ± 0.53 years. Eight patients (28.6%) converted into an overt α-synucleinopathy (iRBD_conv; 3 PD, 3 Dementia with Lewy Bodies—DLB, 1 Multiple System Atrophy—MSA and 1 unspecified atypical parkinsonism); one patient died due to larynx cancer during the second year of follow-up.

### Epigenetic age evaluation

**Fig. 1:**
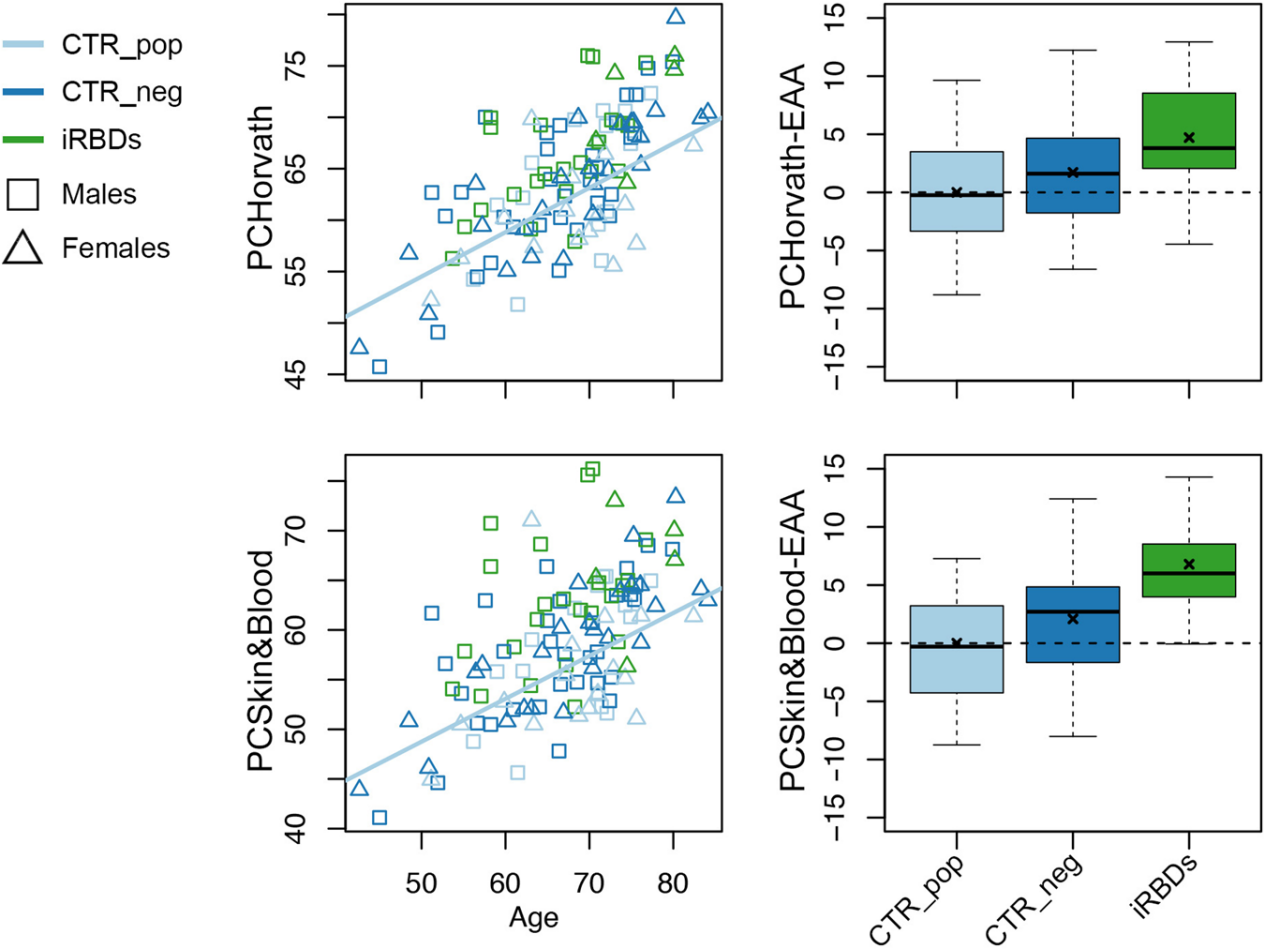
Epigenetic age acceleration in iRBD patients. Left panels: scatter plots of epigenetic age vs chronological age for PCHorvath clock (upper panel) and PCSkin&Blood (lower panel). Right panels: boxplots of PCHorvath-EAA and PCSkin&Blood-EAA for the 3 groups under investigation. x: mean; central line: median; bounds of the box: 25th (lower bound) and 75th (upper bound) percentile; whiskers: minimum (lower whisker) and maximum (upper whisker).

Compared to CTR_neg, iRBDs showed significantly higher PCHorvath-EAA and PCSkin&Blood-EAA values when correcting for experimental batch and sex (Table 1 and Fig. 1). A similar trend was present also for most of the other EAA values, although it did not reach statistical significance (Table 1 and Supplementary Fig. 1).

**Table 1:** Epigenetic Age Acceleration (EAA) measures in the study groups.

	Parameter	CTR_pop (mean ± sd)	CTR_neg (mean ± sd)	iRBDs (mean ± sd)	iRBDs vs CTR_pop *p*-value	iRBDs vs CTR_neg *p*-value
Horvath calculator	Horvath-EAA	0 ± 4.87	0.42 ± 4.78	3.74 ± 5.57	0.189	0.063
	intrinsic-EAA	0 ± 3.04	−0.90 ± 5.92	3.78 ± 6.77	0.285	0.055
	extrinsic-EAA	0 ± 6.77	2.23 ± 5.62	3.78 ± 4.34	0.208	0.381
	Hannum-EAA	0 ± 5.16	1.41 ± 4.34	2.56 ± 3.73	0.286	0.350
	Skin&Blood-EAA	0 ± 3.96	1.07 ± 3.66	1.58 ± 3.44	0.205	0.452
	PhenoAge-EAA	0 ± 6.83	1.92 ± 6.6	3.37 ± 4.62	0.132	0.451
	GrimAge-EAA	0 ± 3.58	−0.63 ± 3.79	1.04 ± 4.41	0.850	0.222
Higgins-Chen calculator	PCHorvath-EAA	0 ± 4.97	1.71 ± 4.55	4.71 ± 4.48	**0.028**	**0.031**
	PCHannum-EAA	0 ± 5.41	1.45 ± 4.40	3.25 ± 4.13	0.083	0.124
	PCSkin&Blood-EAA	0 ± 5.54	2.09 ± 4.73	6.79 ± 6.06	**0.016**	**0.006**
	PCPhenoAge-EAA	0 ± 6.28	2.20 ± 5.32	2.65 ± 4.43	0.133	0.421
	PCGrimAge-EAA	0 ± 2.8	0.39 ± 3.01	2.07 ± 3.01	0.202	0.130
	DunedinPACE	0.92 ± 0.11	0.96 ± 0.10	1.02 ± 0.11	0.131	0.125

*CTR_pop* controls taken from the general population, *CTR_neg* controls negative for REM Sleep Behaviour Disorder (RBD) at videopolysomnography, *iRBDs* isolated RBD patients, *sd* standard deviation. Statistically significant results are in bold.

iRBDs_conv did not significantly differ from non-converted (iRBDs_n_conv) in terms of EAA as measured by the different epigenetic age estimators (Supplementary Table 1). Accordingly, PCHorvath-EAA and PCSkin&Blood-EAA values were still significantly higher in iRBDs compared to CTR_neg also when excluding the 8 iRBDs_conv and the patient died with cancer. Longer disease duration was not correlated with a greater EAA magnitude for any of the estimators (Supplementary Table 2). Finally, no significant differences were observed in estimated blood cell counts when comparing iRBDs and CTR_neg (Supplementary Table 3).

## Discussion

To the best of our knowledge, this is the first work evaluating epigenetic clocks in a cohort of vPSG-confirmed iRBD patients. Epigenetic biomarkers of aging based on genome-wide DNA methylation levels have been evaluated in various fields^6^, including oncology, neurodegenerative conditions, and infectious diseases^7^, where their role as therapy response markers is on study^8^.

Our results suggest that iRBD patients display an accelerated epigenetic aging with respect to iRBD-negative controls. A trend towards increased epigenetic age was evident for almost all the assessed clocks and reached statistical significance for two Principal Component-based clocks (PCHorvath and PCSkin&Blood), possibly because they are less susceptible to technical noise^9^. In iRBD, the increase in EAA could highlight the already concurrent neurodegeneration. Moreover, the evaluation of EAA could shed light on the role of aging^10^ in the α-synucleinopathy neurodegeneration itself, as previously outlined by Horvath^5^, underlying a physiopathological process measurable from blood specimens. For example, aging and PD share basic characteristics such as accumulation of senescent cells, inflammation^11^, and propagation phenomena^2,12^. Supporting this consideration, aging-related changes in the dopamine system seem to approach the biological threshold for parkinsonism, actively producing a vulnerable pre-parkinsonian state^13^. Moreover, neuropathological features of PD can also be found in elderly without clinical signs of parkinsonism, suggesting a continuum between physiological aging and neurodegenerative age-related motor disorders^14^. Similar, though less, evidence links also DLB and MSA to neuroinflammation and accelerated aging^15,16^.

Our data did not show any link between EAA and either conversion to overt α-synucleinopathy or disease duration. Indeed, the limited numerosity of our cohort may have prevented us to grab these relations. However, we can also suppose that there may not be a perfect alignment between the neurodegenerative process itself and the clinical picture of the patients: subjects with more prominent neurodegeneration may exhibit milder clinical signs and may be yet to phenoconvert, similarly for other fluid biomarkers^1^. Moreover, as the neurodegeneration in iRBD has already started, longer duration may not contribute to a further increase in EAA. Eventually, although it is possible to speculate regarding the functional consequences of epigenetic age, the predominantly correlative nature of the study makes it very difficult to distinguish between the clocks as causes, consequences, or passive bystanders of aging^2,4^.

Overall, our study presented several limitations, first, the limited number of patients, which prevented further analyses, and the relatively short duration of clinical follow-up. Moreover, sex distribution was not completely similar, and previous reports showed that males tend to be epigenetically older than females^17^. For this reason, we included sex as covariate in our analysis. Finally, although it is difficult to speculate whether the epigenetic age acceleration observed in blood reflects DNAm alterations occurring in the nervous system, recent studies reported positive blood–brain correlations for different epigenetic clocks^18,19^. Future studies should clarify this point.

In recent years pivotal has been the research of reliable iRBD biomarkers, recently classified depending on their value for diagnosis, progression and prediction of conversion towards α-synucleinopathies^1^. State diagnostic markers differ between groups of iRBD patients and controls, confirming an underlying α-synucleinopathy, and can be considered a proxy of the smoldering neurodegeneration. Within this framework, finding an accelerated epigenetic aging in iRBD would not only increase our knowledge on its contribution in the etiopathology of α-synucleinopathies, but might also give us a measurable state diagnostic biomarker of this condition, without the downside of invasive procedures. Moreover, it may open the path to a putative and easily obtainable way to monitor upcoming disease-modifying therapies, as recently verified for other conditions where accelerated aging plays an important role^8^.

To note, the strength of our results is further supported by the presence of two different control groups, including not only general population controls, but also vPSG-negative subjects, narrowing the potential confounders.

To conclude, increased EAA may be a promising, though yet to be polished, marker of neurodegeneration in iRBD. Indeed, future studies, including longitudinal evaluation of EAA and comparison with other biomarkers, will allow a better understanding of the drivers of these biological clocks and facilitate experiments to test their practical impact on the disorder.

## Methods

### Study participants

All iRBD patients were consecutively recruited from IRCCS Istituto delle Scienze Neurologiche di Bologna (IRCCS-ISNB) between October 2017 and February 2019. The diagnosis of RBD was confirmed by vPSG performed in our center’s sleep laboratory, based on international criteria^20^. We excluded patients with a diagnosis of secondary RBD, such when associated with comorbid neurodegenerative diseases, narcolepsy, structural lesions in the brainstem, or use of drugs with a primary effect on the central nervous system (CNS) (e.g., antidepressants). All iRBDs were consequently followed-up with yearly clinically evaluations until January 2022.

vPSG-negative controls, comparable for age, were recruited among unrelated patients’ caregivers and acquaintances coming to IRCCS-ISNB after the exclusion of concomitant CNS disorders. Controls from the general population included healthy Italian subjects from the PROPAG-AGEING project^21^.

### Epigenetic clocks analysis

Genomic DNA was extracted from whole blood using the QIAmp DNA blood kit (Qiagen) and bisulfite-converted using the EZ DNA Methylation Kit (Zymo Research). Genome-wide DNA methylation was assessed using the Infinium HumanMethylationEPIC BeadChip (Illumina) following manufacturer’s instructions. For Illumina Infinium data preprocessing the minfi Bioconductor package was used to extract signal intensity files. Eight samples had a rate of failed probes (detection p-value > 0.05) higher than 5% and were excluded. Normalization was performed using the preprocessFunnorm function implemented in minfi and probes having a bad detection p-value in more than 1% of the samples (25715) were removed.

Epigenetic age was estimated using 3 different calculators. The first one is the Horvath’s online DNA Methylation Age Calculator (https://dnamage.genetics.ucla.edu/) which returns: (1) the pan-tissue Horvath’s clock; (2) the blood-specific Hannum’s clock; (3) the Skin&Blood clock; (4) the PhenoAge, developed considering clinical measures related to differences in health span and lifespan; (5) the GrimAge, developed considering plasma levels of 7 proteins and smoking pack-years, which is associated with mortality. The second one is the Higgins-Chen calculator^9^, which applies principal component analysis to the five above-mentioned clocks, improving their reliability by minimizing technical noise. The third one is the DunedinPACE clock^22^, derived from the analysis of longitudinal data from individuals from the same birth cohort, which is informative of the rate of age-related deterioration.

For Horvath’s and Higgins-Chen calculators, EAA values were calculated as the residuals of the linear regression between epigenetic age estimates and chronological age, using CTR_pop as the reference group. For Horvath’s calculator, we further estimated the intrinsic and the extrinsic EAA. Intrinsic-EAA, independent from changes in blood cell composition, was calculated correcting Horvath-EAA for estimated white blood cell counts, while extrinsic-EAA, indicative of immunosenescence, was derived by regressing the BioAge4HAStatic value with chronological age, as described in the online tutorial.

Blood cell counts were estimated from DNA methylation data using Horvath’s online DNA Methylation Age Calculator (https://dnamage.genetics.ucla.edu/).

Differences in EAA values, in DunedinPACE and in estimated blood cell counts between CTR_neg and iRBDs or between converted iRBDs (iRBDs_conv) and non-converters (iRBDs_n_conv) were analyzed using type-III analysis-of-variance (ANOVA) correcting for experimental batch and sex. Linear regression was used to calculate the association between EAA values and disease duration, correcting for experimental batch and sex. P-value < 0.05 was retained as significant.

### Standard protocol approvals, and patient consents

We conducted the study according to the Declaration of Helsinki and all participants provided informed written consent. The study was approved by the local ethics committee (no. of approval 79/2015/U/Tess of 15/09/2015 and 16018 of May 2016).

### Reporting summary

Further information on research design is available in the Nature Research Reporting Summary linked to this article.

## Supplementary information

Supplementary Material

REPORTING SUMMARY

## Data Availability

The output from the DNA Methylation Age Calculator is available in Zenodo open-access repository (https://zenodo.org/record/6546165#.YosyIpNBz3A). Anonymized clinical data and metadata will be shared by request from any qualified investigator.
