# Supplementary material for: Epigenetic clocks suggest accelerated aging in patients with isolated REM Sleep Behavior Disorder
Source: NPJ Parkinsons Dis. 2023 Mar 30;9:48. doi: 10.1038/s41531-023-00492-2 (PMC10063653; doi:10.1038/s41531-023-00492-2)
Supplement: Supplementary file 1 — Supplementary Material [file 41531_2023_492_MOESM1_ESM.pdf]

SUPPLEMENTARY MATERIAL

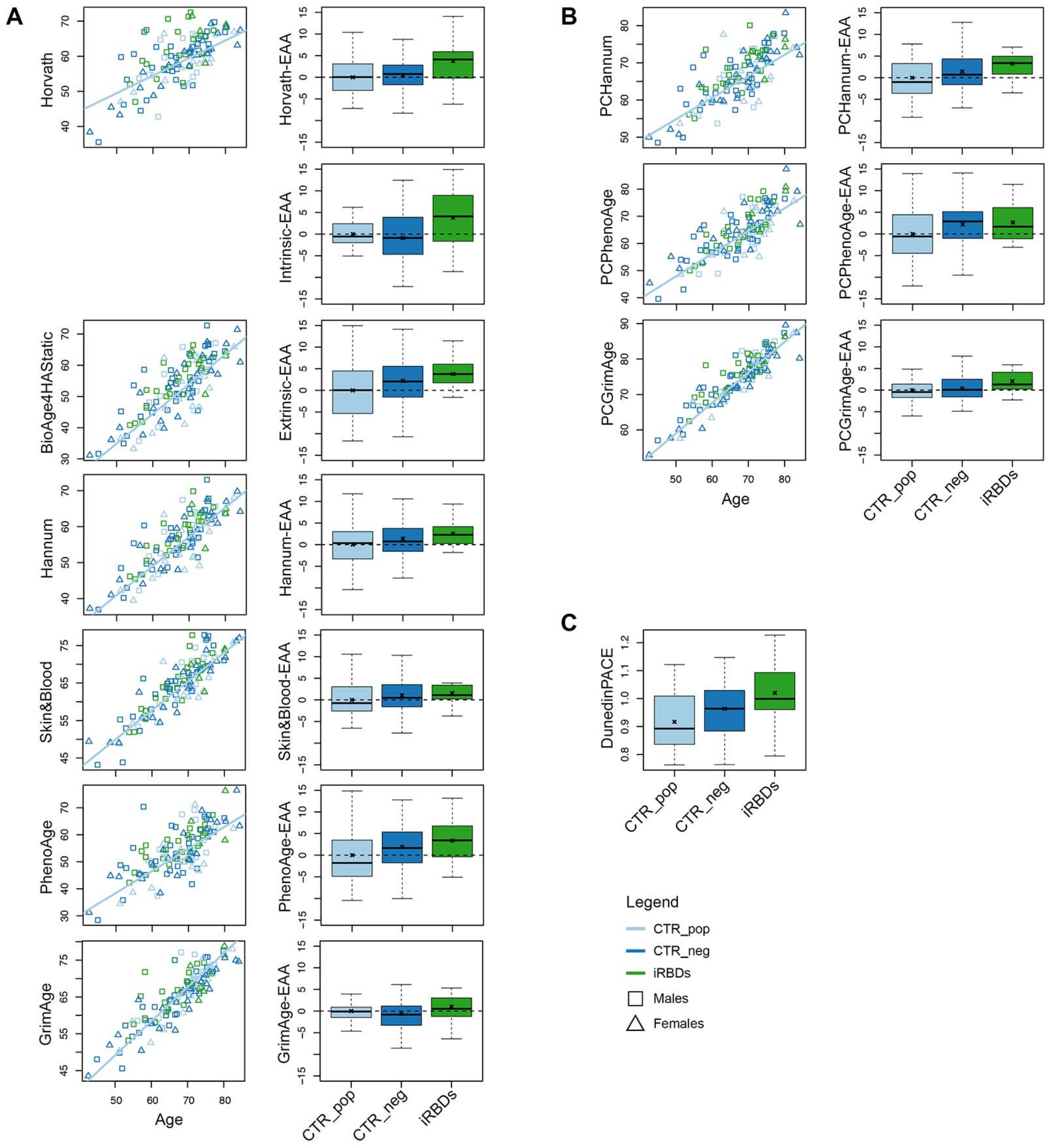

Supplementary Figure 1. Epigenetic age acceleration in iRBD patients. (A) Results from the Horvath's DNA Methylation Age Calculator. Left panels: scatter plot of epigenetic age vs chronological age for the different clocks. Right panels: for each clock, the boxplots of EAA values for the 3 groups under investigation are reported. Note that for Intrinsic-EAA the scatterplot is not reported, as these EAA values were calculated from the Horvath’s clock correcting for estimated blood cell counts. B) Results from the Higgins-Chen calculator. Left panels: scatter plot of epigenetic age vs chronological age for the different clocks. Right panels: for each clock, the boxplots of EAA values for the 3 groups under investigation are reported. Note that for PCHorvath and PCSkin&Blood results are reported in Figure 1. C) Results from the DunedinPACE clock. The boxplots report the values of pace of biological aging for the 3 groups under investigation.

Legend. x: mean; central line: median; bounds of the box: 25th (lower bound) and 75th (upper bound) percentile; whiskers: minimum (lower whisker) and maximum (upper whisker).

Supplementary table 1. Epigenetic Age Acceleration (EAA) measures in converted (or dead) and non-converted iRBD patients.

	Parameter	CTR_neg (mean ± sd)	iRBDs_conv (mean ± sd)	iRBDs_n_conv (mean ± sd)	CTR_neg vs iRBDs_n_conv p-value	CTR_neg vs iRBDs_conv p-value	iRBDs_n_conv vs iRBDs_conv p-value
Horvath calculator	Horvath-EAA	0.42 ± 4.78	1.55 ± 5.73	4.62 ± 5.40	**0.022**	0.677	0.375
	intrinsic-EAA	-0.90 ± 5.92	-0.60 ± 6.13	5.54 ± 6.32	**0.012**	0.945	0.093
	extrinsic-EAA	2.23 ± 5.62	1.39 ± 4.68	4.73 ± 3.92	0.088	0.555	0.098
	Hannum-EAA	1.41 ± 4.34	0.48 ± 3.77	3.39 ± 3.47	0.064	0.479	0.080
	Skin&Blood-EAA	1.07 ± 3.66	0.41 ± 3.62	2.05 ± 3.34	0.103	0.553	0.129
	PhenoAge-EAA	1.92 ± 6.60	1.55 ± 3.90	4.09 ± 4.78	0.235	0.781	0.184
	GrimAge-EAA	-0.63 ± 3.79	-0.87 ± 2.94	1.80 ± 4.73	0.056	0.649	0.18
Higgins-Chen calculator	PCHorvath-EAA	1.71 ± 4.55	4.03 ± 5.81	4.99 ± 3.97	**0.029**	0.243	0.961
	PCHannum-EAA	1.45 ± 4.40	1.71 ± 3.96	3.87 ± 4.13	**0.039**	0.971	0.231
	PCSkin&Blood-EAA	2.09 ± 4.73	4.70 ± 7.36	7.63 ± 5.44	**0.003**	0.198	0.667
	PCPhenoAge-EAA	2.20 ± 5.32	3.33 ± 3.98	2.37 ± 4.67	0.480	0.522	0.779
	PCGrimAge-EAA	0.39 ± 3.01	1.30 ± 2.31	2.37 ± 3.25	0.088	0.611	0.579
	DunedinPACE	0.96 ± 0.10	0.99 ± 0.10	1.03 ± 0.11	0.115	0.501	0.952

Legend. iRBD_conv: isolated REM sleep behaviour disorder patients converted into an overt neurodegenerative disease, or who died, at the end of follow-up; iRBD_n_conv: isolated REM sleep behaviour disorder patients still without any manifest neurodegenerative disease at the end of follow-up. Statistically significant results are in **bold**.

Supplementary table 2. Regression models between Epigenetic Age Acceleration (EAA) measures and iRBD duration.

	Parameter	iRBD duration beta coefficient	iRBD duration p-value
Horvath calculator	Horvath-EAA	0.010	0.488
	intrinsic-EAA	0.009	0.594
	extrinsic-EAA	0.003	0.782
	Hannum-EAA	0.005	0.611
	Skin&Blood-EAA	0.006	0.519
	PhenoAge-EAA	0.004	0.729
	GrimAge-EAA	0.012	0.324
Higgins-Chen calculator	PCHorvath-EAA	0.016	0.174
	PCHannum-EAA	0.014	0.227
	PCSkin&Blood-EAA	0.023	0.107
	PCPhenoAge-EAA	0.008	0.489
	PCGrimAge-EAA	0.006	0.480
	DunedinPACE	0.000	0.969

Legend. iRBD: isolated REM sleep behaviour disorder.

Supplementary table 3. Estimated blood cell types in iRBDs vs CTR_neg patients.

Parameter	CTR_neg (mean ± sd)	iRBDs (mean ± sd)	CTR_neg vs iRBDs p-value
CD8	0.03 ± 0.03	0.04 ± 0.04	0.199
CD4	0.15 ± 0.07	0.12 ± 0.05	0.108
NK	0.06 ± 0.04	0.08 ± 0.04	0.064
B cell	0.04 ± 0.02	0.03 ± 0.02	0.242
Monocytes	0.07 ± 0.02	0.07 ± 0.02	0.270
Granulocytes	0.63 ± 0.09	0.64 ± 0.08	0.695
Plasmablasts	1.76 ± 0.20	1.85 ± 0.21	0.730
CD8pCD28nCD45RAn	9.19 ± 3.19	8.79 ± 3.30	0.448
CD8 naive	218.42 ± 44.87	200.16 ± 35.43	0.215
CD4 naive	617.33 ± 97.64	601.93 ± 122.30	0.184

Legend. iRBDs: isolated RBD patients; CTR_neg: controls negative for REM Sleep Behaviour Disorder (RBD) at videopolysomnography; sd: standard deviation.
