# Supplementary material for: Epigenetic clocks suggest accelerated aging in patients with isolated REM Sleep Behavior Disorder
Source: NPJ Parkinsons Dis. 2023 Mar 30;9:48. doi: 10.1038/s41531-023-00492-2 (PMC10063653; doi:10.1038/s41531-023-00492-2)
Supplement: Supplementary file 2 — REPORTING SUMMARY [file 41531_2023_492_MOESM2_ESM.pdf]

nature portfolio

Corresponding author(s): Federica Provini

Last updated by author(s): Mar 5, 2023

Reporting Summary

Nature Portfolio wishes to improve the reproducibility of the work that we publish. This form provides structure for consistency and transparency in reporting. For further information on Nature Portfolio policies, see our Editorial Policies and the Editorial Policy Checklist.

Statistics

For all statistical analyses, confirm that the following items are present in the figure legend, table legend, main text, or Methods section.

n/a	Confirmed	
☐	☒	The exact sample size (*n*) for each experimental group/condition, given as a discrete number and unit of measurement
☐	☒	A statement on whether measurements were taken from distinct samples or whether the same sample was measured repeatedly
☐	☒	The statistical test(s) used AND whether they are one- or two-sided<br>*Only common tests should be described solely by name; describe more complex techniques in the Methods section.*
☐	☒	A description of all covariates tested
☐	☒	A description of any assumptions or corrections, such as tests of normality and adjustment for multiple comparisons
☐	☒	A full description of the statistical parameters including central tendency (e.g. means) or other basic estimates (e.g. regression coefficient) AND variation (e.g. standard deviation) or associated estimates of uncertainty (e.g. confidence intervals)
☐	☒	For null hypothesis testing, the test statistic (e.g. *F*, *t*, *r*) with confidence intervals, effect sizes, degrees of freedom and *P* value noted<br>*Give P values as exact values whenever suitable.*
☒	☐	For Bayesian analysis, information on the choice of priors and Markov chain Monte Carlo settings
☒	☐	For hierarchical and complex designs, identification of the appropriate level for tests and full reporting of outcomes
☐	☒	Estimates of effect sizes (e.g. Cohen's *d*, Pearson's *r*), indicating how they were calculated

Our web collection on statistics for biologists contains articles on many of the points above.

Software and code

Policy information about availability of computer code

Data collection	On site not-online database accessible upon autentication on password-protected computer.
Data analysis	DNA methylation analysis by Infinium HumanMethylationEPIC BeadChip (Illumina) and data pre-processing are described in Supplementary File 1. Epigenetic age was estimated using 3 different calculators. The first one is the Horvath's online DNA Methylation Age Calculator (https://dnamage.genetics.ucla.edu/) which returns: 1) the pan-tissue Horvath's clock; 2) the blood-specific Hannum's clock; 3) the Skin&Blood clock; 4) the PhenoAge, developed considering clinical measures related to differences in health span and lifespan; 5) the GrimAge, developed considering plasma levels of 7 proteins and smoking pack-years, which is associated with mortality. The second one is the Higgins-Chen calculator,8 which applies principal component analysis to the 5 above mentioned clocks, improving their reliability by minimizing technical noise. The third one is the DunedinPACE clock,9 derived from the analysis of longitudinal data from individuals from the same birth cohort, which is informative of the rate of age-related deterioration.<br>For Horvath's and Higgins-Chen calculators, EAA values were calculated as the residuals of the linear regression between epigenetic age estimates and chronological age, using CTR_pop as reference group. For Horvath's calculator, we further estimated the intrinsic and the extrinsic EAA. Intrinsic-EAA, independent from changes in blood cell composition, was calculated correcting Horvath-EAA for estimated white blood cell counts, while extrinsic-EAA, indicative of immunosenescence, was derived by regressing the BioAge4HAStatic value with chronological age, as described in the online tutorial.<br>Blood cell counts were estimated from DNA methylation data using the Horvath's online DNA Methylation Age Calculator (https://dnamage.genetics.ucla.edu/).<br>Differences in EAA values, in DunedinPACE and in estimated blood cell counts between CTR_neg and iRBDs or between converted iRBDs (iRBDs_conv) and non-converters (iRBDs_n_conv) were analyzed using type-III analysis-of-variance (ANOVA) correcting for experimental batch and sex. Linear regression was used to calculate the association between EAA values and disease duration, correcting for experimental batch and sex. P-value <0.05 was retained as significant.

Supplementary file

DNA extraction and Illumina Infinium analysis
Genomic DNA was extracted from whole blood using the QIAmp DNA blood kit (Qiagen) and bisulfite-converted using the EZ DNA Methylation Kit (Zymo Research). Genome-wide DNA methylation was assessed using the Infinium HumanMethylationEPIC BeadChip (Illumina) following manufacturer's instructions.

Illumina Infinium data preprocessing
The minfi Bioconductor package was used to extract signal intensity files. Eight samples had a rate of failed probes (detection p-value > 0.05) higher than 5% and were excluded. Normalization was performed using the preprocessFunnorm function implemented in minfi and probes having a bad detection p-value in more than 1% of the samples (25715) were removed.

For manuscripts utilizing custom algorithms or software that are central to the research but not yet described in published literature, software must be made available to editors and reviewers. We strongly encourage code deposition in a community repository (e.g. GitHub). See the Nature Portfolio guidelines for submitting code & software for further information.

Data

Policy information about availability of data

All manuscripts must include a data availability statement. This statement should provide the following information, where applicable:

- Accession codes, unique identifiers, or web links for publicly available datasets
- A description of any restrictions on data availability
- For clinical datasets or third party data, please ensure that the statement adheres to our policy

The output from the DNA Methylation Age Calculator is available in Zenodo open-access repository (DOI: https://zenodo.org/record/6546165#.YosylpNBz3A). Anonymized clinical data and metadata will be shared by request from any qualified investigator.

Human research participants

Policy information about studies involving human research participants and Sex and Gender in Research.

Reporting on sex and gender	Our findings apply only to biological sex, which was determined based on self-reporting. Sex-related analysis was performed and represented graphically in the scatterplots present in Figure 1. Self-reported gender data was also recollected, but was not part of this research, which was conducted solely on a biological basis.
Population characteristics	We compared 28 iRBDs (23 males, age 67.92±7.20 years), 57 CTR_neg (32 males, age 66.56±9.56 years) and 31 CTR_pop (15 males, age 68.25±7.23 years); male prevalence was higher among iRBDs (p=0.020), age was comparable (p=0.614). iRBDs presented a mean disease duration of 8.95±6.35 years (range 1 – 28 years) and were followed up for a mean of 3.45±0.53 years. Eight patients (28.6%) converted into an overt α-synucleinopathy (iRBD_conv; 3 PD, 3 Dementia with Lewy Bodies – DLB, 1 Multiple System Atrophy – MSA and 1 unspecified atypical parkinsonism); one patient died due to larynx cancer during the second year of follow-up.
Recruitment	All iRBD patients were consecutively recruited from IRCCS Istituto delle Scienze Neurologiche di Bologna (IRCCS-ISNB) between October 2017 and February 2019. The diagnosis of RBD was confirmed by vPSG performed in our center's sleep laboratory, based on international criteria. We excluded patients with a diagnosis of secondary RBD, such when associated with comorbid neurodegenerative diseases, narcolepsy, structural lesions in the brainstem, or use of drugs with a primary effect on the central nervous system (CNS) (e.g., antidepressants). All iRBDs were consequently followed-up with yearly clinically evaluations until January 2022.<br>vPSG-negative controls, comparable for age, were recruited among unrelated patients' caregivers and acquaintances coming to IRCCS-ISNB after exclusion of concomitant CNS disorders. Controls from the general population included healthy Italian subjects from the PROPAG-AGEING project.
Ethics oversight	We conducted the study according to the Declaration of Helsinki and all participants provided informed written consent. The study was approved by the local ethics committee (no. of approval 79/2015/U/Tess of 15/09/2015 and 16018 of May 2016).

Note that full information on the approval of the study protocol must also be provided in the manuscript.

Field-specific reporting

Please select the one below that is the best fit for your research. If you are not sure, read the appropriate sections before making your selection.

☒ Life sciences ☐ Behavioural & social sciences ☐ Ecological, evolutionary & environmental sciences

For a reference copy of the document with all sections, see nature.com/documents/nr-reporting-summary-flat.pdf

Life sciences study design

All studies must disclose on these points even when the disclosure is negative.

Sample size	Sample size calculation was based on the recruiting centre recruiting force, in order to ensure a smooth enrolment.
Data exclusions	All iRBD patients were consecutively recruited from IRCCS Istituto delle Scienze Neurologiche di Bologna (IRCCS-ISNB) between October 2017 and February 2019. The diagnosis of RBD was confirmed by vPSG performed in our center's sleep laboratory, based on international criteria. We excluded patients with a diagnosis of secondary RBD, such when associated with comorbid neurodegenerative diseases, narcolepsy, structural lesions in the brainstem, or use of drugs with a primary effect on the central nervous system (CNS) (e.g., antidepressants). All iRBDs were consequently followed-up with yearly clinically evaluations until January 2022.<br>vPSG-negative controls, comparable for age, were recruited among unrelated patients' caregivers and acquaintances coming to IRCCS-ISNB after exclusion of concomitant CNS disorders. Controls from the general population included healthy Italian subjects from the PROPAG-AGEING project.
Replication	Validated laboratory standards were used in order to ensure reproducibility.
Randomization	Not relevant for sthe study as it is a prospective non-pharmacological study.
Blinding	Not relevant for sthe study as it is a prospective non-pharmacological study.

Reporting for specific materials, systems and methods

We require information from authors about some types of materials, experimental systems and methods used in many studies. Here, indicate whether each material, system or method listed is relevant to your study. If you are not sure if a list item applies to your research, read the appropriate section before selecting a response.

Materials & experimental systems

n/a	Involved in the study	
☒	☐	Antibodies
☒	☐	Eukaryotic cell lines
☒	☐	Palaeontology and archaeology
☒	☐	Animals and other organisms
☒	☐	Clinical data
☒	☐	Dual use research of concern

Methods

n/a	Involved in the study	
☒	☐	ChIP-seq
☒	☐	Flow cytometry
☒	☐	MRI-based neuroimaging
